# ^13^C-NMR Assessment of the Pattern of Organic Matter Transformation during Domestic Wastewater Treatment by Autothermal Aerobic Digestion (ATAD)

**DOI:** 10.3390/ijerph6082288

**Published:** 2009-08-19

**Authors:** Anna V. Piterina, John Barlett, J. Tony Pembroke

**Affiliations:** 1 Department of Chemical and Environmental Sciences, Material and Surface Science Institute, University of Limerick, Limerick, Ireland; E-Mail: anna.piterina@ul.ie; 2 Centre for Sustainability, Sligo Institute of Technology, Sligo, Ireland; E-Mail: bartlett.john@itsligo.ie

**Keywords:** autothermal thermophilic aerobic digestion (ATAD), ^13^C-NMR, biosolids, biodegradation, organic matter transformation

## Abstract

The pattern of biodegradation and the chemical changes occurring in the macromolecular fraction of domestic sludge during autothermal thermophilic aerobic digestion (ATAD) was monitored and characterised via solid-state ^13^C-NMR CP-MAS. Major indexes such as aromaticity, hydrophobicity and alkyl/O-alkyl ratios calculated for the ATAD processed biosolids were compared by means of these values to corresponding indexes reported for sludges of different origin such as manures, soil organic matter and certain types of compost. Given that this is the first time that these techniques have been applied to ATAD sludge, the data indicates that long-chain aliphatics are easily utilized by the microbial populations as substrates for metabolic activities at all stages of aerobic digestion and serve as a key substrate for the temperature increase, which in turn results in sludge sterilization. The ATAD biosolids following treatment had a prevalence of O-alkyl domains, a low aromaticity index (10.4%) and an alkyl/O-alkyl ratio of 0.48 while the hydrophobicity index of the sludge decreased from 1.12 to 0.62 during the treatment. These results have important implications for the evolution of new ATAD modalities particularly in relation to dewatering and the future use of ATAD processed biosolids as a fertilizer, particularly with respect to hydrological impacts on the soil behaviour.

## 1. Introduction

Autothermal Thermophilic Aerobic Digestion (ATAD) has increasingly been used worldwide for tertiary treatment of animal manure, domestic wastewater and for food, pulp and textile wastes [1–4]. The thermophilic conditions that occur during the ATAD process facilitate microbial biomass production [1,5–7] and degradation of multiple substrates in the sludge [3,4]. The elevated temperature also facilitates improved degradative kinetics which in turn is complemented by efficient mixing and aeration during the process [6]. The process is energy-efficient, environmentally friendly and economical because the heat required is generated by bacterial metabolism, few chemical additions are needed, and the final product can be readily applied to land without management restrictions for pathogen control (US EPA Regulation 503). Significant progress has been made in the optimization and adaptation of ATAD technology since it was first introduced in the early 1970s. Most of the research pertaining to ATAD digesters has focused on laboratory-scale process performance [1,8], total solid removal efficiency [2], optimisation of the ATAD process for treatment of difficult to process wastes [3,4,9] and the dewatering characteristics of the resultant biosolids [10,11]. Continuous innovation of the process is shown by the growth of the number of patents for “next” generation ATAD technologies [12].

Driven by metabolically active and heat-producing micro-organisms the efficiency of ATAD treatment depends on the ability of the sludge to be degraded and transformed due to enzymatic activities of resident microorganisms. Little is known about the discrete compositional changes and overall fate of wastewater organic matter during such thermophilic digestion. Thus monitoring and characterization of the organic matter (OM) component of sludge at different stages of the ATAD process may help to improve our understanding of this advanced wastewater treatment process. Knowledge of the biochemistry behind the degradation and the patterns by which degraded materials are generated during each intermediate step would allow for a rational approach to the optimisation of the treatment process and offer the ability to tailor the treatment process to a particular feed or final product quality and composition.

Domestic waste is normally comprised of waste from toilets, sinks, garbage disposal, dishwashers, baths, showers and washing machines. It can also contain waste from septic tanks, portable toilets, sanitation devices or similar systems that receive only domestic waste. It will also include material of animal or plant origin, kitchen cleaning chemicals, detergents, fecal materials [13]. Sludge following primary and secondary treatment also contains bacterial cellular constituents (aliphatic straight-chain lipids and peptidoglycans), volatile fatty acids,’ and non-biodegradable or recalcitrant material originating from traces of plastic, cotton, toilet paper and plant material originally associated with the waste.

Solid state ^13^C-Nuclear Magnetic Resonance has been used in an effort to determine *in situ* both qualitatively and semi-quantitatively the molecular features of the organic matter and it has been identified as a promising tool in a majority of environmental studies in the area of wastewater analysis and composting processes [3,14–27]. The benefit of this non-destructive technique lies in its sensitivity, independence of sample solubility, and the ability to perform analysis at a variety of temperatures. To distinguish between the protonated and non-protonated carbon pool in ATAD sludge, we used cross polarization magic angle spinning (CP-MAS) with and without interrupted decoupling (non-quaternary suppression (NQS) [28,29].

The goal of the present study was to analyse the changes of chemical and spectroscopic properties of sewage sludge composed of a mixture of primary and secondary pre-treated sludges of domestic origin, during the ATAD process. Characterization of the distribution, degradation and evolution of the major chemical groups that occurred during tertiary treatment under aerobic thermophilic conditions was carried out to follow the biodegradation processes, and evaluate the characteristics of the final product as a land fertilizer.

## 2. Material and Methods

### 2.1. Description of the Site and Sample Collection

Sludges were sampled from the Killarney Sewage Treatment Works, which processes the majority of the domestic wastewater from a population of 20,000–51,000 people in Killarney, Co Kerry, Ireland. The mean solid production by the treatment plant approaches 500 tonnes per annum. The plant is unique in that the feed for the ATAD process is composed of a mixture of primary and secondary treated sludges, which are further thickened to 4–6% total solids (TS) on a belt filter. The daily feed rate for the ATAD process is in the range of 15–30 m^3^/d. The aerobic thermophilic digestion takes place via a two-reactor semi-batch process with the processed sludge stored in a holding tank and allowed to go anaerobic. ATAD reactors 1A and 2A (110 m^3^) are operated in series, with the partially digested sludge being fed from the first ATAD (operating temperature ranges of 35–49 °C), to the second ATAD, where the operation temperature is in the range of 58–63 °C. The treated sludge from the ATAD reactors is stored in one of three holding tanks (275 m^3^), where the sludge is cooled due to the absence of aeration which results in a cessation of aerobic microbial activity. A schematic of the plant, with detailed information on its operational parameters can be found in earlier papers [7,12]. To compare the outputs of the two ATAD reactors where aeration and mixing are provided and the holding tank which is unmixed and essentially anaerobic a sampling strategy as outlined below was adopted. Sampling was carried out at five different stages of the composting process. Sample 1 was collected after the belt filter and classified as a representative of the digester’s feed. Sample 2 was taken from Reactor 1A, 2 hours after the start of mesophilic digestion. Sample 3 was collected from Reactor 2A, 2 hours after the transfer of mesophilic sludge and the start of the thermophilic digestion. Sample 4 was taken from Reactor 2A, 24 hours after start of the thermophilic digestion and 30 minutes before completion of digestion and prior to transfer into the final holding tank. Samples 5 and 6 are taken from the holding tank after storage for 1 and 9 days, respectively. The retention time for the whole ATAD process is 9 days (storage time not included). Other chemical characteristics such as pH, total solids (TS), volatile solid (VS) content and temperature values were determined for each sample. Enumeration of viable bacteria present in the sludge at each sampling point were determined using a combination of Live/Dead Bacterial viability stain and epifluorescence microscopy as described by the manufacturer (Invitrogen, UK).

### 2.2. Sample Preparation for NMR Analysis

Sludge samples were freeze-dried under conditions shown previously to be optimal for preservation of such heterogeneous organic matter [30]. After lyophilisation, samples were ground into a powder but not pre-treated further.

### 2.3. Solid state Nuclear Magnetic Resonance Spectroscopy

**Figure 1.**
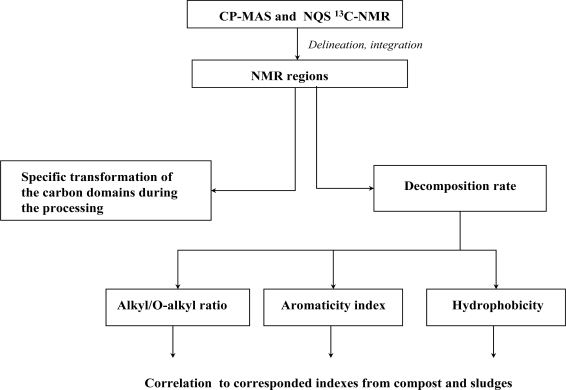
Schematic representation of steps for obtaining the various datasets (in boxes) discussed in the text. Obtained datasets are presented and compared in paragraphs 3.1–3.6.

The experimental layout of this study is presented in Figure1.

#### 2.3.1. NMR acquisition conditions and processing parameters

^13^C-NMR spectra were obtained in solid state using the same conditions optimized for quantitative comparisons between spectra of lignocellulosic and humic substances [19,24,31]. The spectrometer used was a Bruker MSL 100 (2.35 T) operating at 25.1 MHz for ^13^C-NMR. Magic angle spinning was performed at 13 MHz. The recycle delay of the CP-MAS pulse sequence was set to 1.5 s. Cross polarization contact time was 1 ms. The spectral width was 125 kHz and the acquisition time was 20 ms. In total, 3,000 scans (sludge samples prepared from feed, reactor 1A and product) and 16,000 scans (for sludge samples from thermophilic reactor 2A), were acquired and accumulated for each resonance spectrum.

For non-quaternary suppression the pulse sequence made use of a dephasing time of 100 ms. This dephasing time was enough to suppress the signals of the protonated carbons, thereby revealing only the quaternary ones. MesreC V4.3.6.0. Software was utilised to analyse data. After Fourier transformation, a zero order phase correction and a Multipoint Baseline Correction were applied to process the spectra and the chemical shift was calibrated to tetramethylsilane (=0 ppm) [28].

#### 2.3.2. Spectral analysis and peaks assignment

The determination of spectral peaks, assigned spectral interpretation and preliminary assignations in chemical shifts were carried out as described previously [3,14–27]: 0 to 48 ppm, alkyl C; 48 to 60 ppm, methoxyl C; 60 to 93 ppm, O-alkyl C; 93 to 112 ppm, di-O-alkyl and some aromatic C; 112 to 140 ppm, aromatic C–C and C–H; 140 to 160 ppm, phenolic C; and 160 to 190 ppm, carboxyl-C (including amides and esters). Further details and the numerical order of these assignations of chemical shifts are combined in Table 2 and Table 3. The areas of the chemical shift regions were determined from the integral curves and were expressed as a fraction of total area (relative intensity, which in this study is also designated index, e.g., alkyl index). As several factors influence the intensity distributions in CP-MAS NMR spectra [31,32], the relative intensities were only used for comparative purposes.

### 2.4. Evaluation of the Specific Transformation of Different Types Changes in Different C-Domains during Different stages of the ATAD Process

The values of the relative intensities after different steps of ATAD processing were utilised to estimate and observe the changes in the major C-domains at different stages of the ATAD process. Given the various stages of ATAD processing characterised by unique physico-chemical parameters (Table 4) [7,12] to estimate behavior of the each C-domain during the ATAD process, changes for each C-domain (mean value calculated from the spectra) and decomposition indexes were calculated. The following formula was applied as described previously by Smernik *et al.* [22]:

(1) $$\begin{array}{l} {}[\text{Transformation}\;\text{of}\;\text{the}\;\text{C}-\text{domain}\;\text{occurring}\;\text{during}\;\text{the}\;\text{processing}\;\text{stage}=\\ (\%\;\text{relative}\;\text{intensity}\;\text{of}\;\text{the}\;C-\text{domain}\;\text{in}\;\text{the}{\;}^{13}C-\text{NMR}\;\text{spectra}\;\text{of}\;\text{the}\;\text{sample}\;\text{obtained}\;\text{at}\;\text{the}\;\text{end}\\ \text{of}\;\text{the}\;\text{processing}\;\text{stage})-(\%\;\text{relative}\;\text{intensity}\;\text{of}\;\text{the}\;C-\text{domain}\;\text{in}\;\text{the}{\;}^{13}C-\text{NMR}\;\text{spectra}\;\text{of}\;\text{the}\\ \text{sample}\;\text{obtained}\;\text{the}\;\text{start}\;\text{of}\;\text{the}\;\text{processing}\;\text{stage}) \end{array}$$

The changes were plotted to help visualize of the evolution of the C-domains during each step and presented in the section “Results and Discussion”.

### 2.5. Extent of the Decomposition Rate

To estimate of the degradation of the sludge and estimate the potential of sludge maturation we utilized decomposition indices [16,34]. Based on the integrated areas of C types from the ^13^C-NMR spectra, we determined the following ratios among the organic groups present: aromaticity (Equation 2) [34], alkyl/O-alkyl ratios (Equation 3) [16] and hydrophobicity (Equation 4) [16]. These are used as common indexes of humification and decomposition rates of organic matter during composting and sludge treatment.

(2) $$\text{Aromaticity}=\frac{\text{Aromatics}}{\text{Alkyl}+O-\text{alkyl}+\text{aromatic}}\times 100\%$$

(3) $$\frac{O-\text{alkyl}}{\text{alkyl}}\;\;\text{ratio}=\frac{O-\text{alkyls}}{\text{alkyls}}$$

(4) $$\text{Hydrophobicity}=\frac{\text{Aromatic}+\text{alkyls}}{\text{Carbonyl}/\;\text{Acyl}+O-\text{alkyl}}$$

## 3. Results and Discussion

### 3.1. Physico-Chemical Characteristics Obtained at Different Stage of the ATAD Process

Physico-chemical characteristics of sludge samples obtained from different stages of the ATAD process are summarized in Table 3. A reduction in TS from 6.3% to 4.2% occurred gradually during the ATAD treatment and occurs in all reactors during ATAD processing (Table 3). A reduction in VS of 39.2% occurred after processing in reactor 1A and Reactor 2A, which is satisfactory for EPA requirements. Removal of the volatile solid were more pronounced in Reactor 1A (26.1%), with less reduction occurring during processing in reactor 2A (16%), a further reduction on 3.6% took place during the storage. The digestion process is characterised by a continuous increase in pH of the sludge from pH6.3 (feed) to pH 9.1 (Reactor 2A, 24 hours). This pH change has previously been described to correspond to the rate of biodegradation and hydrolysis of protein-based material followed by further deamination of the peptide and amino acid products with subsequent release of ammonia [35]. Due to limited microbial transformation of ammonia at elevated temperatures [36], the released ammonia is collected in the “gas-off scrubber” installed on site plant (peak levels may reach over 1,000 ppm), some becomes solubilised and accumulates in the bulk sludge water of the reactor contributing to the increase in pH [1,5]. Thus during the overall process the feed changes from a slightly acidic pH to alkali values as the temperature increases from 17 to 63 °C. The decrease in total solids results from biodegradation and heat and gas release during the ATAD stages (Table 3). The colour of the feed also changes from grey to grey-brown during treatment in reactor 1A (mesophilic thermophilic stage), and further changes to dark brown after treatment in the thermophilic stage (reactor 2A).

The distinct pattern of sludge colour change during the processing is most probably due to a Maillard reaction, where condensation of amino groups with carbohydrates occur and results in visible absorbance shifts [37]. More details on seasonal temperature fluctuation profiles of the ATAD processing stages and data on the reduction of volatile and total solids content during the Killarney ATAD wastewater plant have been presented [7,12]. CTC-reduction assays were not used in this study for evaluation of respirometric index or activity of the microbial community, due to potential interference by factors in the sludge samples and to its low correlation at elevated temperatures. Instead enumeration of viable bacteria was performed using the Live/Dead Bacterial Viability staining assay. As may be seen from Table 3 viability was not compromised by the increase in temperature. High viable numbers were observed in reactor 1A, which decreased initially in the early stages in Reactor 2A. This was possibly due to temperature shock of mesophilic species before thermophilic or thermoduric species adapted to the higher temperatures. After this adaptive phase the number of viable bacteria increased substantially to numbers similar to those observed in reactor 1A.

### 3.2. Spectra Characterisation and Peak Assignments

**Figure 2.**
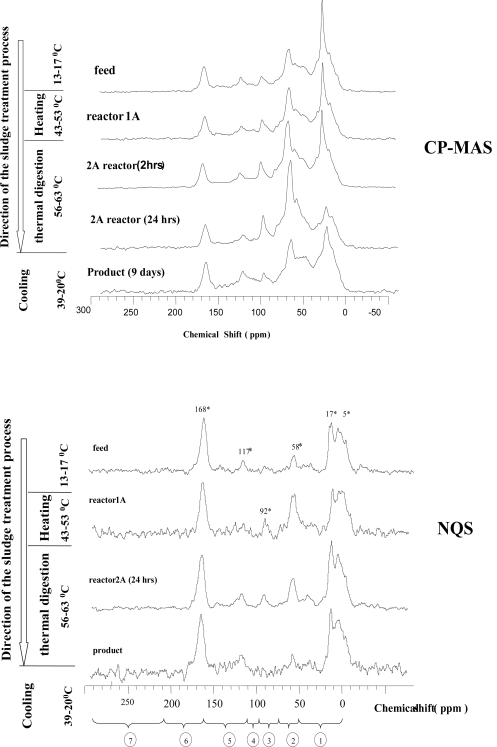
Cross polarization and magic angel spinning (CP-MAS) 13C-NMR and Non-Quaternary suppression spectral editing (NQS) spectra of sludge material at different stages of ATAD processing. Temperature and the process steps are shown as a bar on left side of the chart. Axes representing chemical shift are shown in ppm.

Carbon-13 NMR was used to access the general composition of carbon types during ATAD processing. The resulting resonance spectra acquired with cross polarisation and magic angle spin with spectral editing for removal of signal intensities from non-protonated carbon (NQS) are presented on the Figure 2 Integrated values for each C-domain can be found in Table 4. As can be observed in Figure 2, the spectra of the feed, product and mesophilic and thermophilic sludges were characterized by the presence of several signals in the area of paraffinic carbon in alkyl chains (0 and 50 ppm), aliphatic carbon substituted by oxygen and nitrogen and including the methoxyl groups of aromatic ethers (50 and 110 ppm) and double bonded or aromatic carbon (110 and 160 ppm). The changes in these C types caused by the ATAD treatment processes can be quantified by integrating the peak areas of the spectra (Table 4).

*Aliphatic C (0–45 ppm)* region is assigned to proteins, lipids, and aliphatic branched and short chained molecules [33]. Peaks in the alkyl-area in all spectra obtained showed as wide shoulder. This can be explained as being a contribution of a mixture of aliphatic molecules with different structures. Several distinct peaks are recognized however. We observed several peaks at *17 ppm* (as part of the narrow shoulder in Figure 2), *10 ppm* (short aliphatic C), *23 ppm* (branched aliphatic C), *30 ppm* (the methylenic C in the long chains of aliphatic compounds) (Table 2). The alkyl region (46-0 ppm) showed the maximum at *30 ppm*, for polyethylene carbons found in lipids and lipid polymers and support a large contribution by lipid polyesters. Major changes were observed in the *23 ppm* peak relative intensity which corresponded to β -carbon of branched aliphatic chained compounds (Table 2). A small peak or shoulder at approximately *21 ppm* in the CP-MAS can be observed, which is frequently in the literature attributed to acetate groups in hemicelluloses [20,21].

In the NQS spectra (Figure 2) peaks at 17 ppm and 5 ppm were observed corresponding to short-branched aliphatic molecules, which changed during processing (the decrease only occurs in the 17 ppm resonance peak, and shoulder in the alkyl region become more diffuse).

*O and N-alkyl C (45–110 ppm) region* is assigned primarily to O-substituted alkyl carbon in carbohydrates, but also includes methoxyl carbon and N-substituted alkyl carbon in protein. Figure 5 shows that signal at 76 ppm and 83 ppm are the predominant signals in these regions. These signals, derived from hemicelluloses, are generally contained within the cellulose peaks [20,21]. Their intensities increase as the ATAD temperatures increase, and reach their highest value after digestion in the thermophilic step. After prolonged storage we observed a decline in intensity of this particular peak.

*The aromatic region (160–110 ppm)* can be divided into a region between 160–140 ppm for aromatic carbons linked to O or N and between 140–110 ppm for non-substituted and C-substituted aromatic carbons. The spectral regions between 140–160 ppm have been assigned to lignin, phenols, aromatic ethers or amine moieties [16]. In this area only 1 peak was observed in all spectra obtained at between 117–120 ppm.

*The carboxylic region (160–200 ppm)* can also be divided into two sub regions, one between 160 ppm and 184 ppm for carboxylic acids (–COOH), mainly organic acid that are free or involved in esters or amides, and a second between 184 ppm and 200 ppm for the carbonyl group (–C-O) present in aldehydes, ketones and organic acid [33]. In this region, we observed one peak at 168ppm in the NQSs and CP-MAS spectra in all ATAD sludge samples.

*Carbonyl C region (184–210)* ^13^C-NMR spectra for sludges at all stages of ATAD appeared to lack the carbonyl-C resonances in this region which would indicate the absence of such chemical groups as ketones, aldehydes and organic acids [16,20,21].

### 3.3. Evaluation of the Specific Transformation in Different Carbon Domains during the overall ATAD Process

**Figure 3.**
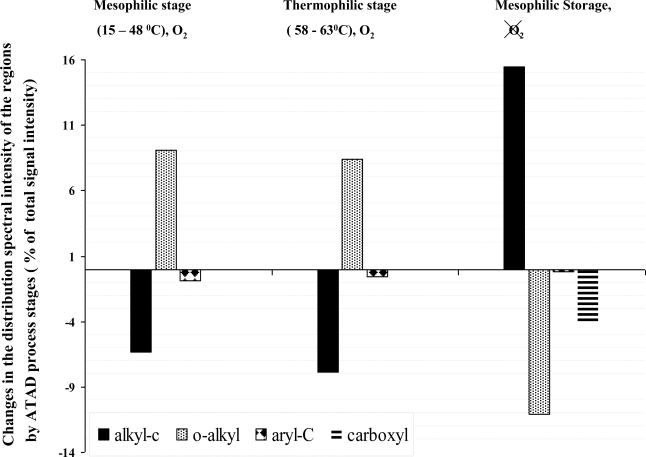
Specific transformation of different C-domains observed by solid-state ^13^C Cross Polarization (CP) nuclear magnetic resonance (NMR) and spectral intensities of the sludge samples collected at different stages of the ATAD process. Operating conditions relating to oxygen and temperature ranges during the ATAD processing steps are shown on the top of the plot. The mean values of the data utilized to perform comparison are presented in Table 4 and calculation carried out as recommended by Smernik *et al.* [20,21] and described in section “Materials and Methods”.

The evolution of the alkyl-C, O-alkyl, aryl-C, carboxyl –C domain during each stage of the ATAD process was calculated as the difference of the integrated intensity values of this region from the spectra taken for sludges. The pattern of changes is presented in Figure 3. During the self-heating stages of the process operating in the mesophilic and thermophilic range of temperatures profiles showed a clear tendency of increase in the O-alkyl region (overall increase in intensity 16.2%), and a decline in the intensity of the alkyl–region (14.2%). The degradation pattern of the aliphatic compound are most likely due to the enzymatic activity of the inhabiting microorganisms and showed slightly higher degradation (7.8%) compared to the mesophilic reactor (6.2%). No difference was observed in the carboxyl –C domain during the aerobic high temperature stage. There was a slight decline observed in the amount of aryl-C detected during the mesophilic and thermophilic stages. After storage the alkyl region can be characterized as a broad-range peak, which represents a variety of short and long branched members of the alkyl-C family. It is impossible to separate the contributions of protein or lipid to the C- alkyl region, and while there is some reduction in the proportion of long- CH_2_ chains, overall both lipid and protein C decrease in the biosolids during processing. We observed new protein accumulating during the storage stage which may be due to anaerobic biosynthesis. During the storage of sludge in the holding tank for 9 days, dramatic changes in all C-domains were observed compared to the fresh biosolids obtained at the end of the thermophilic processing. A dramatic increase in the alkyl-region (15.3%), a decrease in the O-alkyl C (11.9%), with no changes in the aromatic region was observed with a decrease in the value of the intensity for the carboxyl-C domain. This behaviour of the organic domain was directly opposite to what we observed in the self-heating mesophilic and aerobic thermophilic reactor most likely due to neo-synthesis of the organic material and the development of new biomass.

### 3.4. Extent of the Decomposition Rate

**Figure 4.**
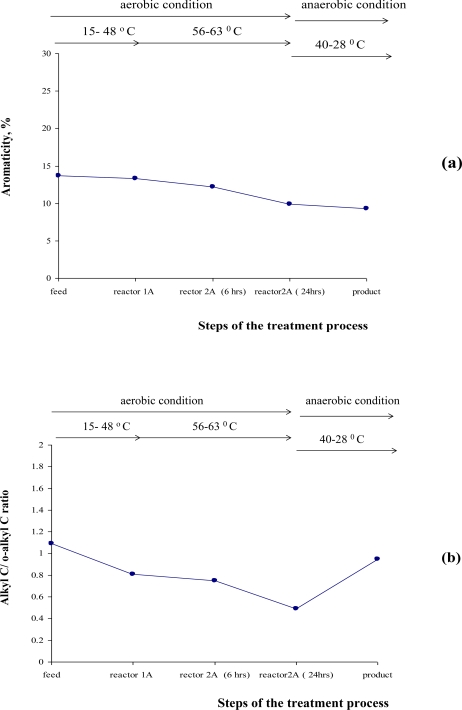
Evolution of the decomposition indexes of the sludge during the ATAD process. (a) Graphical plot of the aromaticity values, (b) ratios of the relative intensities of the carbon domains detected in the alkyl (0–55 ppm) and O-alkyl (55–110 ppm) regions for ^13^C-NMR spectra obtained for sludge samples during ATAD processing. The mean value of the data utilized to perform calculations is presented in Table 2. Whole sludge samples were analysed by ^13^C-NMR with cross polarizations and magical angle spinning techniques. Operating condition related to oxygen and temperature range during the process steps are shown on the top of the plot. Aromaticity values and Alkyl/O-alkyl ratios was calculated as described [16,34].

Based on the integrated areas of the C types from the ^13^C-NMR spectra, the following ratios among the organic groups were also calculated: aromaticity (Equation 2), alkyl/O-alkyl ratios (Equation 3) and hydrophobicity (Equation 4) as common indexes of humification and decomposition rates of organic matter during composting of sludge. The following indexes were plotted to show overall change of the decomposition indices (Figure 4). From the data we can see that the feed sample can be characterized with the highest aromaticity index (14%), aromaticity values decrease slightly during the mesophilic stage (13%), decline again over the thermophilic stage (10.1%), and then remain constant during the storage (9.9%). The alkyl/O-alkyl ratio was highest (1.1%) in the feed (Figures 4 and 5), during the start of the self-heating process its value decreased. During the mesophilic stage this value only slightly decreased, but during the high temperature treatment phase the value decreased and reached 0.43%. Upon storage the value increased up to 8.4% possibly due to neo-synthesis of the protein-rich EPS components by bacterial communities present. With respect to hydrophobicity indexes (Figure 4), we observed that the original feed values were 1.12%, these decreased during the self-heating stage to 0.68%, and decrease even more during the storage step to 0.48%. The decomposition indexes were also compared Table 6.

Thus a distinctive set of chemical modifications and transformations of organic matter was observed during ATAD treatment of domestic sludge in this study. It is evident that the molecular structure of fresh treated sludge entering ATAD differs from the structure of the organic matter of samples taken at the end of the process. The inlet sludge had the strongest aliphatic properties and as ATAD digestion occurs and the temperatures increase we observed changes to certain domains of organic matter as a consequence of increased metabolic activity. During the thermophilic process stage we observed significant changes in lipid and protein with the accumulation of carbohydrate-type organic material (Figures 2–4). We observed a clear change in 0-alkyl organics over alkyl, carbonyl, carboxyl and aromatic group organics. The growth and activity of metabolically active microorganisms inhabiting the mesophilic Reactor 1A, Reactor 2A (after 4 hrs) and thermophilic organisms in Reactor 2A most likely contribute significantly to these changes. Such activities are supported by previous studies which showed the presence of bacterial isolates with high proteolytic activity [1]. The decline in the intensity of the aliphatic C-domain may be attributed to the degradability of compounds with this group by the microbial community inhabiting the early stage reactors (reactor 1A operated at mesophilic to low thermophilic temperatures). An increase of the O-alkyl domain occurred during processing and exhibited maximum values after the thermophilic stage (Figures 4 and 5). This may be due to the accumulation of soluble products of the degradation of cellulose rich material derived mainly from residual plant and paper material which remained only partially degraded following earlier primary and secondary treatments. The cellulose component of the sludge may lose its crystallinity due to the slight increase in alkalinity which results from solubilised ammonia and due to the enzymatic activity of the dynamic microbial population. The presence of non-crystalline cellulose was in fact detected by microscopic analysis and staining which support these observations (data not shown). Therefore, the decrease of the crystallinity of the cellulose and alterations in the hemicellulose is indicative of cellulose biodegradation during the ATAD process. Microbial products and cell-wall components of Gram-positive bacteria such as *Bacillus* and *Clostridium sp*. [1,7] may also contribute to the intensity of the O-alkyl region [43], which is most predominant in the ^13^C-NMR spectra of the thermophilic sludge. These organisms have been found to be the predominant microorganisms able to grow and proliferate at ATAD operating conditions and temperature [1,7]. Since the non-protonated Alkyl C and O-alkyl C domains did not undergo any major transformation these groups must be contained within compounds highly resistant to degradation. We believe that this may give a new insight on the possible reasons for the poor dewaterability of ATAD biosolids, which is known to depend on the ratio of the protein to carbohydrate domains in the sludge [40]. The low index of hydrophobicity and low ratio of aliphatic compounds to carbonaceous compound is an important factor in predicting floc dissociation and may indeed be a reason for the low settleability of ATAD sludge [10,40,41]. During the storage of the sludge following ATAD processing, where the conditions can be characterised as anaerobic and mesophilic, new products are formed as observed in the NMR spectra. Mobile and rigid alkyl C-groups are prominent at this stage and the final ATAD sludge begins to resemble that for a typical mesophilic digested sludge as described in the literature [39,42]. In relation to humification and stabilisation indexes, analysis of the ATAD samples revealed a low intensity aromatic region corresponding to lignins, phenols, aromatic ethers or amine moieties (11%), this is present at all stages of the ATAD process and decreases with processing to 7.8% in relative intensity (Table 5). Based on our analysis the calculated index of the hydrophobicity and alkyl/O-alkyl ratio may be more sensitive indexes of the progress of the ATAD process, as changes appeared to correlate with process development whereas the aromaticity indexes did correlate with the stages of the ATAD process or the extent of sludge digestion. Condensation and generation of aromatic biomolecules or polymers did not appear to take place in the thermophilic sludge. A high degradation of mobile carbon in the alkyl-region and accumulation of the soluble products of microbial activity such as hydrolysis products of cellulose, were observed. The low Alkyl-to-O-alkyl ratio in the thermophilic sludge (Table 6) may be interpreted as the comparatively low degradation rate of carbohydrate polymers compared to that of protein or lipidic material.

The highly alkyl nature (and specifically alkyl with high molecular mobility) of stored biosolids can influence the fate and behavior of sewage sludge applied to agricultural land [39,42]. The absence of highly condensed aromatic organic structures, the absence of humification, and the presence of alkyl and O-alkyl groups can be beneficial in application of biosolids for soil management and as a rapid remedy for soils exhausted of organic material. The full environmental impact of the application of sludge rich in O-alkyl carbon organic matter derived from ATAD on soil health has not been analysed but would need to be addressed should long term soil application of ATAD treated biosolids be carried out.

## 4. Conclusions

Solid-state ^13^C-NMR was used to determine the changes of chemical and spectroscopic properties of sewage sludge of municipal origin during a 9 day ATAD process and following 9 days of storage. The carbon distribution determined from ^13^C-NMR spectra can be used for quantification of the conversion of the organic matter following thermophilic biological processing and sludge stabilization.

Our data indicates notable variations in the spectra between the initial sludge at inlet and its treated counterpart following 9 days of thermophilic digestion and following storage. The main spectral differences were observed in the O-alkyl region (0–50 ppm), in the C-O-alkyl/N-alkyl region (50–110 ppm) providing information on the alterations occurring in the different biochemical entities, chiefly lipids and carbohydrates. The original feed organic matter gave a distinctive NMR spectrum containing a large amount of alkyl carbon. The sub spectra were distinctively alkyl-rich and close to a microbial biomass spectra which may be due to the large increase in microbial biomass occurring during the ATAD process and responsible for the large increase in temperature.

Fresh biosolids was rich in alkyl C as previously reported [20–22,32] whereas thermophilic ATAD sludge exhibited a very low aromaticity index compared to other composted products previously reported and also had a high o- Alkyl/alkyl ratio decomposition value.

Prolonged storage of the treated biosolids under essentially anaerobic mesophilic conditions for 9 days also altered the sludge characteristics making it resemble a traditional sludge. Thus it would be important to analyse the true impact of storage conditions on the quality of the ATAD sludge. Because of the change over to a potentially anaerobic population at the storage stage the metabolic capabilities of such a population may make recalcitrant material for the thermophilic stage a substrate for such an anaerobic population. We are addressing this by examining the exo-enzyme activities present upon storage and comparing this to that present at other stages of ATAD. Additional studies are needed to quantify the effects of different pre-treatment techniques on the ATAD sludge and whether augmentation by various thermophilic microorganisms as supplements could affect the rate and extent of degradation of recalcitrant or poorly degraded materials as certain parts of the alkyl-C compounds for example did not show any degradation. Such fractions need to be identified and characterised, in order to improve ATAD process performance. The nature of the organic matter present may have an important impact on dewatering parameters while the nature of the domains present may also impact soil amendment policy and influence hydrological properties of the soil.

## Figures and Tables

**Table 1.** Chemical shift region of 13C-NMR spectra and assignment to the corresponding organic domains. Data was compiled from the following references [3,14–27,33].

**Organic domain**		**Chemical shift region (ppm)**
**alkyl**	Aliphatic carbons (**–CH3, –CH2**) (Biopolymers, lipid, proteins)	0–55
***O*-alkyl**	Methoxyl **(–OCH3)**	45–72
	**C–O** of carbohydrate-type compounds; **C-2, C-3, C-4, C-5** and **C-6** non-crystalline component of the cellulose	72–97
	Anomeric carbon of polysaccharides and **C-1** of the cellulose or xylan or aromatic lignins)	97–118
**aryl**	Unsubstituted and alkyl-substituted aromatic-**C**; Aromatic lignins	110–140
	Lignins, phenols Aromatic ethers or amines moieties	140–165
**carboxyl**	Carboxyl-**C** in aliphatics and aromatics groups; **C** - in amidic groups	165–205

**Table 3.** Characteristics of the sludge samples obtained at different stages of ATAD treatment.

**Obtained samples**	**T,°C**	**pH**	**TS, %**	**VS, %**	**Color**	**Viable bacterial number (Live/Dead Viability assay)**

Feed (primary +secondary sludge)	11	6.3	6.3	82,3	grey	(7.3 +0.4) × 10^6^
Reactor 1A	43	7.0	5.8	56.2	grey -brown	(8.2 +0.1) ×10^11^
Reactor 2A (2hrs)	53	8.1	5.1	59.1	Brown	(4 + 0.2) × 10 ^8^
Reactor 2A (24 hrs)-Fresh biosolids	63	9.1	4.2	43.1	dark brown	(4.9+ 0.1) ×10 ^11^
Product-(9 day storage in holding tank)	14	7.8	4.6	39.5	grey-Brown	(6.2+ 0.3) ×10^6^

**Table 4.** Integration values for the major organic C-type domains in the CP-MAS (CP) and NQS ^13^ C- NMR spectra of sludge samples obtained from different stage of ATAD biosolids processing *(values shown as % of the integration value of total C).* Chemical shifts are shown in part per million units (ppm).

	**CP-MAS**						
	**Chemical shift regions (ppm)**						
	205-165	165-140	140-118	118-97	97-72	72-55	55-0
	Carbon Distribution (%)						
Feed	8.9	4.4	6.6	6.8	17.4	14.0	41.9
Reactor 1A	8.1	4.1	6.7	7.9	20.4	14.3	38.5
Reactor 2A (2hrs)	7.0	3.7	6.4	8.5	23.4	15.4	35.6
Reactor 2A (24hrs)	7.0	3.8	5.7	9.7	29.2	16.8	27.7
Product (9days)	2.9	0.7	7.6	5.9	21.2	18.5	43.2
	**NQS**						
	**Chemical shift regions (ppm)**						
	205-165	165-140	140-118	118-97	97-72	72-55	55-0
	Carbon Distribution (%)						
Feed	26.6	3.5	5.0	2.7	7.5	5.4	49.4
Reactor 1A	18.6	3.0	6.8	3.9	10.9	6.3	50.4
Reactor 2A (2hrs)	19.7	3.9	5.8	4.7	11.9	6.4	47.6
Reactor 2A (24hrs)	21.6	3.7	3.2	2.6	17.8	7.9	43.2
Product (9days)	24.5	6.3	7.5	0.7	6.4	5.1	49.4

**Table 6.** Comparison of aromaticity (arom), hydrophobicity (hyd), and alkyl-to-O-alkyl ratios for ATAD from this study and sludges, composts and soils from a variety of the origins reported in the literature. The mean values of the data utilized to perform calculation are presented in Table 2. Aromaticity values were calculated a as described previously [34]. Alkyl/O-alkyl ratio was calculated as described previously by Baldock *et al*. [16].

**Source of the material**	**Decomposition Indexes**			**Refs**
	Arom,%	A/O-Alkyl ^(b)^	Hydr	

**Sludges and manures**				
Pig faeces	10	0,57	0,64	[17]
Primary settling tank	20.5	–	–	[17]
Secondary treatment	28.7	–	–	[17]
Pulp industry	–	0.44	–	[15]
Raw Cattle manure	–	0.27	–	[38]
Composted Cattle manure	–	0.56	–	[38]
Composted mixture (faeces + straw)	15	0.23	0.39	[38]
Straw	06	0.15	0.29	[38]

**ATAD**				
Feed	14	1.1	1.12	This study
Fresh Biosolids	10.4	0.48	0.62	This study
Biosolids (9 days storage)	9.7	0.9	0.48	This study

**Soil**				
Farmed sludge soil	13	0.29	0.40	[19]
Planted forest litter	11	0.35	0.31	[39]
